# Extending the UTAUT model to explore the acceptance and use of generative AI: the roles of generative AI identity and trust

**DOI:** 10.3389/fpsyg.2026.1846181

**Published:** 2026-07-31

**Authors:** Der-Fa Chen, Han-Piao Lin, Tzu-Hsin Chu

**Affiliations:** 1 Graduate Institute of Technological and Vocational Education, National Changhua University of Education, Changhua City, Taiwan; 2 Department of Electrical and Mechanical Technology, National Changhua University of Education, Changhua, Taiwan; 3 Department and Graduate Institute of Information Management, Yu Da University of Science and Technology, Miaoli County, Taiwan

**Keywords:** adult learning, generative AI identity, generative AI trust, generative artificial intelligence (generative AI), multi-group analysis

## Abstract

**Introduction:**

Generative AI (GenAI) technology has been rapidly integrated into diverse domains, including education. It has fundamentally reshaped the global learning landscape, exerting a significantly positive influence on students’ learning experience. However, the factors influencing adult learners’ behavioral intention to use GenAI have not yet been fully understood. Therefore, this study aims to explore key factors influencing the behavioral intention to use GenAI among adult learners from central Taiwan. Based on the unified theory of acceptance and use of technology (UTAUT) model, this study integrated two constructs—GenAI identity and trust—to extend the model.

**Methods:**

A structured questionnaire survey was performed to collect data from 718 adult learners who were aged 50 or above and from central Taiwan. Partial least squares (PLS) and Partial least squares-Multi-group analyses (PLS-MGA) were employed for data analysis.

**Results:**

The following results were obtained: (1) social influence and facilitating conditions are significant antecedent factors influencing adult learners’ behavioral intention to use GenAI—conversely, performance expectancy has a negative influence on such intention; (2) GenAI identity and trust are key predictive variables of performance expectancy and effort expectancy; (3) different gender groups exhibit significant differences in most structural paths of the research model.

**Discussion:**

These findings provide theoretical support for context-sensitive strategies in adult learning and the educational application of GenAI.

## Introduction

Lifelong learning is a significant component of adult life in modern society (Gorges, 2025). Since most adult learners can independently decide the type and level of learning activities they will engage in, learning tools play a central role in adult learning. When an extra learning mode brings nothing but learning burdens to an adult learner, they tend to experience frustration or demotivation (McKenna et al., 2020; van der Stap et al., 2024). Adult learners typically possess strong intrinsic learning motivation and high autonomy. Meanwhile, they face diverse demands from work, family, and personal responsibilities (Amoozegar et al., 2024; Kim et al., 2025). Artificial intelligence (AI) has become a core driver for the development of modern society, which fundamentally reshapes the way we work, learn, and communicate. Rapidly evolving generative AI (GenAI), in particular, has become an important technology and key driving force for a new technological revolution (Gazit et al., 2026; Xiaomin et al., 2025). In recent years, GenAI has swiftly penetrated and become integrated into various domains, such as the healthcare sector (Trang et al., 2025), education (Yakubu et al., 2025), business (Kim et al., 2024).

In the education sector, GenAI has fundamentally reshaped the global learning landscape. AI chatbots, such as ChatGPT, Gemini, Copilot, and DeepSeek, and text-to-visual generation models, such as DALL-E, are all viewed as bringing positive contributions to improved work efficiency and enhanced ease of use (Kim et al., 2024). Traditional skill-oriented learning is no longer sufficient to satisfy society’s demand for talent. Specifically, GenAI has been extensively applied in the field of education. By designing personalized learning pathways, fostering the production of interdisciplinary knowledge, and employing simulated scenarios, a learner-centered instructional environment is constructed to enhance learners’ engagement and learning outcomes (Xiaomin et al., 2025; Yakubu et al., 2025). Therefore, GenAI technology consistently drives innovation across diverse fields, with its scope of application steadily expanding and its developmental potential continually increasing. However, the adoption of GenAI in adult learning remains highly limited, highlighting an urgent need to explore factors influencing the employment of this technology in adult learning.

Despite the enormous potential applications of GenAI, few empirical studies have examined adult learners’ usage intentions concerning AI. Thus far, multiple theoretical models have been proposed to predict technology usage behavior. Among these models, the Technology Acceptance Model (TAM) and the Unified Theory of Acceptance and Use of Technology (UTAUT) model are the two most representative and most frequently employed fundamental theoretical frameworks for investigating user technology acceptance (Chao, 2019; Fareed and Kirkil, 2025; Jin and Rui, 2026). The UTAUT model proposed by Venkatesh et al. (2003) is one of the most influential theoretical frameworks used to explain user behavior concerning information technology and information systems. It focuses on user behavior regarding information technology and explains users’ technology acceptance intention (Fareed and Kirkil, 2025; Jin and Rui, 2026; Venkatesh et al., 2003). This model regards performance expectancy (PE), effort expectancy (EE), social influence (SI), and facilitating conditions (FC) as primary factors predicting user behavioral intention to use information systems or technologies. The effects of these factors are moderated by age, gender, experience, and voluntariness of use (Fareed and Kirkil, 2025; Jin and Rui, 2026; Venkatesh et al., 2003; Wang et al., 2025) The UTAUT model has been applied to diverse fields, including e-learning, education, chatbots, and healthcare demonstrating strong effectiveness (Chao, 2019; Fareed and Kirkil, 2025; Su and Chao, 2022; Trang et al., 2025; Wang et al., 2025; Yakubu et al., 2025).

Previous studies have proposed multiple mechanisms to facilitate the use of AI and relevant supportive behavior (Liu X. S. et al., 2022; Lv et al., 2022). One key approach consists in promoting individual AI identity (Carter et al., 2020). AI identity has been widely viewed as a construct with such positive effects as reducing perceived threats to personal identity and enhancing job performance (Alahmad and Robert, 2020; Cao et al., 2023). As GenAI tools become increasingly integrated into educational contexts, understanding their effects on learners’ cognition and usage intention has become ever more important, given their significant potential to enhance work efficiency and creativity (Kim et al., 2024; Song et al., 2026; Zhang and Yu, 2025). Based on previous studies’ accounts of AI usage and supportive behavior using AI identity and given the increasingly wider adoption of GenAI in education, the present study attempted to extend the notion of AI identity and propose the preliminary concept of “generative AI identity” (GenAIID) with a discussion of its influence.

With the rapid diffusion of GenAI tools (e.g., ChatGPT and Gemini), public trust in GenAI has become a key factor influencing its developmental trajectory (Thaiduong, 2026). Previous research observes that trust helps to reduce uncertainties and risks commonly encountered in the advancement of technology (Kolesar and Galbraith, 2000). It is viewed as a significant antecedent condition for predicting technology acceptance, particularly in the application contexts of emerging technologies such as AI. This viewpoint has been supported by diverse theoretical frameworks for technology adoption including the UTAUT (Tanantong and Wongras, 2024; Thaiduong, 2026; Zhang and Yu, 2025). As GenAI is steadily integrated into daily lives, trust in GenAI not only reduces perceived risks and uncertainties but also plays a key role in promoting acceptance.

Although research has been conducted to comprehensively examine the application of GenAI in education, few studies have focused on adult learners. GenAI technology possesses immense potential for improving the learning outcomes of adult learners. Nevertheless, no research has explored factors that influence adult learners’ intention to use GenAI tools. In addition, the widespread application of GenAI in education is still hindered by technical, sociocultural, and institutional barriers, which correspond to the core constructs of the UTAUT, such as social influence and facilitating conditions (Xiaomin et al., 2025). To address this research gap, this study aims to investigate adult learners’ behavioral intention to adopt GenAI through the UTAUT framework. To optimize the framework, an extended model was established by integrating GenAI identity and GenAI trust (GenAITR) in order to more comprehensively and effectively explain adult learners’ decision-making processes concerning the use of GenAI, thereby gaining deeper insights into factors influencing adult learners’ behavioral intention. The objectives of this study are as follows: (1) to explore antecedent factors influencing adult learners’ behavioral intentions concerning GenAI usage; (2) to examine the role of GenAI identity in influencing performance expectancy and effort expectancy; and (3) to investigate the role of GenAI trust in influencing performance expectancy and effort expectancy. This study makes several contributions to the literature. First, it examines diverse factors that affect the behavioral intention to use GenAI based on the UTAUT model. Second, by incorporating GenAI identity and trust into the UTAUT model, this study extends the framework to better understand adult learners’ behavioral intentions of using GenAI.

## Literature review

### Adult learning and generative artificial intelligence

Unlike conventional higher education and children’s education, adult learning predominantly corresponds to adult learners’ life experience, social roles, and complex responsibility structures. Adult learners typically juggle work, family, and social responsibilities while occupying different life stages and fulfilling other obligations. Consequently, their learning motivation is often closely linked to career development, professional advancement, lifelong learning, and self-actualization (Green, 2024; Loeng, 2018; Rodrigo et al., 2024). Furthermore, adult learners are often characterized by intrinsic learning motivation, high autonomy, and abundant life experience that enables self-direction and self-management (Amoozegar et al., 2024). Adult learning theory has sought to explain why teaching approaches catering to adult learners’ educational requirements and learning characteristics are more effective than general learning approaches in satisfying adult learners’ needs. In addition, adult learners typically need to accommodate diverse needs concerning work, family, and personal commitments while they pursue education and learning activities.

In this context, GenAI tools show enormous potential for providing support (Kim et al., 2025). By using conversational human–machine interaction, GenAI reshapes traditional information search and knowledge production (Zhang and Yu, 2025). Unlike early AI tools that were primarily used to analyze existing materials, GenAI systems can create new texts, images, and codes, blurring the boundary between human and machine creation (Thaiduong, 2026). GenAI relies on large-scale training data and deep-learning algorithms, enabling it to meet users’ needs for conversational interaction and knowledge acquisition (Zhang and Yu, 2025). In recent years, GenAI has been rapidly integrated into educational systems and has fundamentally reshaped global educational and learning experience. Widely recognized for its capacity to enhance learning outcomes and support personalized learning (Kim et al., 2024), it is also expected to further revolutionize traditional adult learning practice by offering tailored learning experience. GenAI tools, such as ChatGPT, Gemini, Copilot, DeepSeek, InstructGPT, and DALL-E, can tailor instructional strategies to adult learners’ needs in order to enhance learning effectiveness. The application of GenAI facilitates enhanced learning efficiency, fosters critical thinking, and provides learning support to learners with learning disabilities or other special learning needs (Kim et al., 2024; Xiaomin et al., 2025; Zhang and Yu, 2025).

### Theory of acceptance and use of technology (UTAUT)

Over the past few years, researchers have developed diverse theoretical models to explore factors influencing individual technology usage. One of the most widely employed fundamental theories in the discussion of user technology acceptance is the UTAUT (Chao, 2019; Fareed and Kirkil, 2025; Jin and Rui, 2026). The UTAUT was proposed by Venkatesh and his colleagues Morris, Davis, and Davis in 2003 (Venkatesh et al., 2003). t is an effective theoretical framework that aims to explain and predict individual technology acceptance and usage (Fareed and Kirkil, 2025; Jin and Rui, 2026; Li and Wei, 2025; Venkatesh et al., 2003; Yakubu et al., 2025). Venkatesh et al. (2003) reviewed and synthesized eight classical models and theories in the field of information system acceptance, which originated from social psychology, information system management, and behavioral psychology. After analyzing the common characteristics and limitations of these frameworks, the researchers proposed the UTAUT (Venkatesh et al., 2003).

The eight models and theories synthesized into the UTAUT model are the Theory of Reasoned Action (TRA), Theory of Planned Behavior (TPB), Technology Acceptance Model (TAM), a model combining TAM and TPB (C-TAM–TPB), a model of PC utilization (MPCU), Social Cognitive Theory (SCT), a Motivational Model (MM), and Innovation Diffusion Theory (IDT) (Chao, 2019; Fareed and Kirkil, 2025; Jin and Rui, 2026; Su and Chao, 2022; Trang et al., 2025; Yakubu et al., 2025). The UTAUT model addresses the limitations of the TAM and provides a more comprehensive analysis of users’ intentions to adopt technology. The model has developed into one of the most influential theoretical models in the field of information system and information technology acceptance. It predicts users’ behavioral intention to adopt an information technology or system by examining the direct effects of four core constructs (i.e., performance expectancy, effort expectancy, social influence, and facilitating conditions). The effects of these factors on behavioral intention are moderated by socio-demographic variables including age, gender, experience, and voluntariness of use (Chao, 2019; Venkatesh et al., 2003; Wang et al., 2025).

In this study, performance expectancy (PE) refers to the degree to which adult learners believe that using GenAI will enhance their performance in specific tasks. This construct has been repeatedly proven to be the strongest factor in predicting the intention to use technological devices (Gazit et al., 2026; Xiaomin et al., 2025; Yakubu et al., 2025). Effort expectancy (EE) refers to adult learners’ perceived ease of use, which is the cognitive load and difficulty they perceive when learning and using GenAI. Social influence (SI) refers to the degree of influence from important others (e.g., family, friends, colleagues, or supervisors) on adult learners’ attitudes and behaviors toward using GenAI. Facilitating conditions (FC) refer to adult learners’ perceived availability of necessary infrastructure and support when using GenAI. Finally, behavioral intention (BI) is also a core construct in technology adoption models, which refers to the degree to which adult learners expect to use GenAI frequently in the future.

The UTAUT model has been widely applied in studies of various technological domains, including artificial intelligence-generated content (AIGC) tools, m-learning, e-learning systems, English teacher training, and chatbots (Chao, 2019; Fareed and Kirkil, 2025; Jin and Rui, 2026; Su and Chao, 2022; Trang et al., 2025; Wang et al., 2025; Yakubu et al., 2025). Wang et al. (2025) established a theoretical framework combining the UTAUT and Task-Technology Fit (TTF) models to discuss key factors influencing design students’ adoption of AIGC tools. Their findings demonstrated that performance expectancy, effort expectancy, and social influence significantly enhance students’ usage intention, with social influence exerting the strongest effect. By contrast, facilitating conditions were found to have no influence on usage behavior. Jin and Rui (2026) examined GenAI literacy and acceptance among English as a foreign language (EFL) teachers by integrating the UTAUT model with self-determination theory. Their findings showed that GenAI cognitive ability positively influenced all dimensions of UTAUT as well as behavioral intention (BI). In addition, all UTAUT dimensions, including performance expectancy, effort expectancy, social influence, and facilitating conditions, positively predicted behavioral intention. Fareed and Kirkil (2025) combined the TAM and the UTAUT model to explore the effects of human–computer interaction on students’ usage intention and perceived success regarding the adoption of an e-learning system. The results identified facilitating conditions, effort expectancy, perceived ease of use, and perceived usefulness as primary predictors. Previous studies have shown that performance expectancy, effort expectancy, social influence, and facilitating conditions are driving factors influencing behavioral intention (Chao, 2019; Su and Chao, 2022; Tanantong and Wongras, 2024; Trang et al., 2025; Yakubu et al., 2025). Some studies (Su and Chao, 2022; Tanantong and Wongras, 2024) have demonstrated that effort expectancy has a significantly positive influence on performance expectancy. In light of the foregoing analysis, this study proposes the following hypotheses:

*H1*: Performance expectancy has a positive influence on adult learners’ behavioral intention to use GenAI.

*H2*: Effort expectancy has a positive influence on adult learners’ behavioral intention to use GenAI.

*H3*: Social influence has a positive influence on adult learners’ behavioral intention to use GenAI.

*H4*: Facilitating conditions have a positive influence on adult learners’ behavioral intention to use GenAI.

*H5*: Effort expectancy has a positive influence on adult learners’ performance expectancy toward GenAI usage.

### External constructs

Although the UTAUT model provides a solid theoretical foundation, the rapid evolution of GenAI warrants extending the model to address its limitations. Therefore, this study incorporated GenAI identity and trust into the model to explore how these two constructs operate in the field of adult learning. Specifically, this study focused on adult learning in Taiwan and analyzed how GenAI identity and trust exert an influence through integration with the UTAUT model, thereby further enhancing the predictive power of the model in explaining adult learners’ GenAI adoption behavior.

### Generative AI identity

Identity is a significant mechanism underlying individuals’ sustained self-perception and also has positive effects on individuals’ self-evaluation and self-verification abilities (Burke and Stets, 2009). Identity theory elucidates how social structures influence individuals, shape their understanding of self-identity, and affect the behaviors they adopt in specific social contexts, thereby explaining their behavioral performance (Reychav et al., 2019; Stets and Biga, 2003). Within this framework, individual behavior is determined by the degree to which individuals identify with various objects, roles, and characteristics in a group.

On the basis of identity theory, scholars have gradually extended the concept of identity to the adoption of information technology and AI, positing that these technologies are not merely functional tools but are also likely to be internalized as part of the self-concept. Accordingly, notions such as information technology identity (IT identity) and AI identity have been proposed (Cao et al., 2023; Nash, 2024). IT identity arises from an individual’s desire to extend their self-concept (Aron et al., 2003). It also denotes the self-concept and perceptions formed through the relationship between the individual and IT, which encompasses their beliefs, attitudes, and behaviors during technology usage and human-technology interaction (Nash, 2024; Agarwal and Karahanna, 2000). Agarwal and Karahanna (2000) remarked that when individuals evaluate a specific technology positively and believe that the technology has valuable features that can enhance their ability and improve their task performance, their IT identity will be strengthened. Alahmad and Robert (2020) argued that a holistic understanding of IT identity helps to advance research on AI identity; they proposed a preliminary concept of AI identity that was based on the notion of IT identity to understand how individuals’ identification with AI systems affects their work performance. Cao et al. (2023) redefined AI identity, observing that it is individuals’ incorporation of AI-related knowledge, skills, and abilities into their self-concept as central components of their self-definition.

The fast-growing GenAI technology has rapidly been integrated into and revolutionized the global education and learning landscape, receiving recognition for its potential to enhance learning outcomes and support individualized learning (Kim et al., 2024). Therefore, GenAI identity has emerged as a significant research topic in the academic community. This study observes that when adult learners positively evaluate GenAI, they believe that GenAI possesses valuable features that can enhance their abilities and learning effectiveness, which will thereby strengthen their GenAI identity. Based on the definitions of IT identity and AI identity proposed by previous studies (Alahmad and Robert, 2020; Cao et al., 2023), this study conceptualizes GenAI identity as a context-specific identity construct in the generative AI environment. Cao et al. (2023) defined AI identity as the extent to which individuals incorporate AI-related knowledge, skills, and abilities into their self-concept as central components of their self-definition. While this definition provides an important theoretical foundation, generative AI differs from AI more broadly in several theoretically meaningful ways. Conventional AI systems are commonly designed for prediction, classification, recognition, recommendation, automation, or decision support. In contrast, generative AI systems are characterized by natural language interaction, iterative dialogue, content generation, contextual responsiveness, and human–AI co-creation. These properties enable users to interact with generative AI not merely as an external technological tool, but as a cognitive and creative partner that participates in learning, writing, ideation, knowledge synthesis, and problem solving. Accordingly, this study defines GenAI identity as the extent to which adult learners incorporate GenAI-related knowledge, skills, creative practices, and problem-solving capabilities into their self-concept and role perception through their ongoing relationship with generative AI. When adult learners develop high levels of GenAI identity, GenAI becomes an extension of their job role and self-identity rather than merely an external tool used at work, thereby enhancing the likelihood of proactive GenAI adoption and GenAI-related supportive behavior.

Previous research notes that identity is an important external factor influencing perceived usefulness and perceived ease of use in the TAM, and it has a more significant influence on perceived usefulness (Chonsalasin et al., 2025). Fu et al. (2025) observed that social identity has a direct influence on perceived usefulness. In conclusion, identity is a critical external influencing factor of perceived usefulness and perceived ease of use. However, current research has not examined whether GenAI identity is a significant external factor influencing performance expectancy and effort expectancy in the UTAUT model. The UTAUT model is a development of the TAM, and performance expectancy and effort expectancy in the UTAUT model are derived from the notions of perceived usefulness and perceived ease of use, respectively, in the TAM. Previous research has observed that AI identity is a construct with positive effects and is capable of enhancing work performance (Alahmad and Robert, 2020; Cao et al., 2023). Based on the above analysis, this study posits that GenAI identity will exhibit positive effects on performance expectancy and effort expectancy. Thus, the following hypotheses are proposed.

*H6*: GenAI identity has a positive influence on adult learners’ performance expectancy toward GenAI usage.

*H7*: GenAI identity has a positive influence on adult learners’ effort expectancy toward GenAI usage.

### Generative AI trust

Trust is viewed as a key factor that facilitates information system usage. As trust plays a central role in the adoption of emerging technologies such as AI, people in various domains have increasingly realized the potentially profound influence of AI (Choung et al., 2023; Tanantong and Wongras, 2024). This leads to greater emphasis on building trustworthy AI systems. With the rapid advancement of AI technologies, products and services developed using AI are increasingly subject to individuals’ trust, as trust plays a crucial role during the early stages of new technological development (Chi et al., 2023; Liu X. et al., 2022). AI trust refers to individuals’ favorable attitude and confidence toward AI during their interaction with AI based on their belief that AI has the ability to help them achieve their goals (Zhang and Yu, 2025; Zhang et al., 2025). With the rapid advancement and extensive application of GenAI, when users are highly confident about their ability to use and understand GenAI, they are more likely to recognize the reliability of GenAI and the results it generates, which will further enhance their trust on GenAI. GenAI trust has gradually become a new research topic of interest in both academic and practical contexts. Based on the definition of AI trust, this study defines GenAI trust as adult learners’ favorable attitudes and confidence toward GenAI during their interaction with GenAI, including their belief that GenAI has the ability to help them achieve their goals.

Empirical studies in diverse fields, such as AI human resources recruitment systems (Tanantong and Wongras, 2024), and mobile payments (Khalilzadeh et al., 2017), have established that trust is a significant antecedent factor of performance expectancy and effort expectancy (Khalilzadeh et al., 2017). Tanantong, and Wongras (2024) revealed that trust in AI technology has significantly positive effects on performance expectancy and effort expectancy. It is, therefore, reasonable to assume that GenAI trust has positive effects on performance expectancy and effort expectancy toward GenAI. The following hypotheses are proposed:

*H8*: GenAI trust has a positive influence on adult learners’ performance expectancy toward GenAI usage.

*H9*: GenAI trust has a positive influence on adult learners’ effort expectancy toward GenAI usage.

**FIGURE 1:**
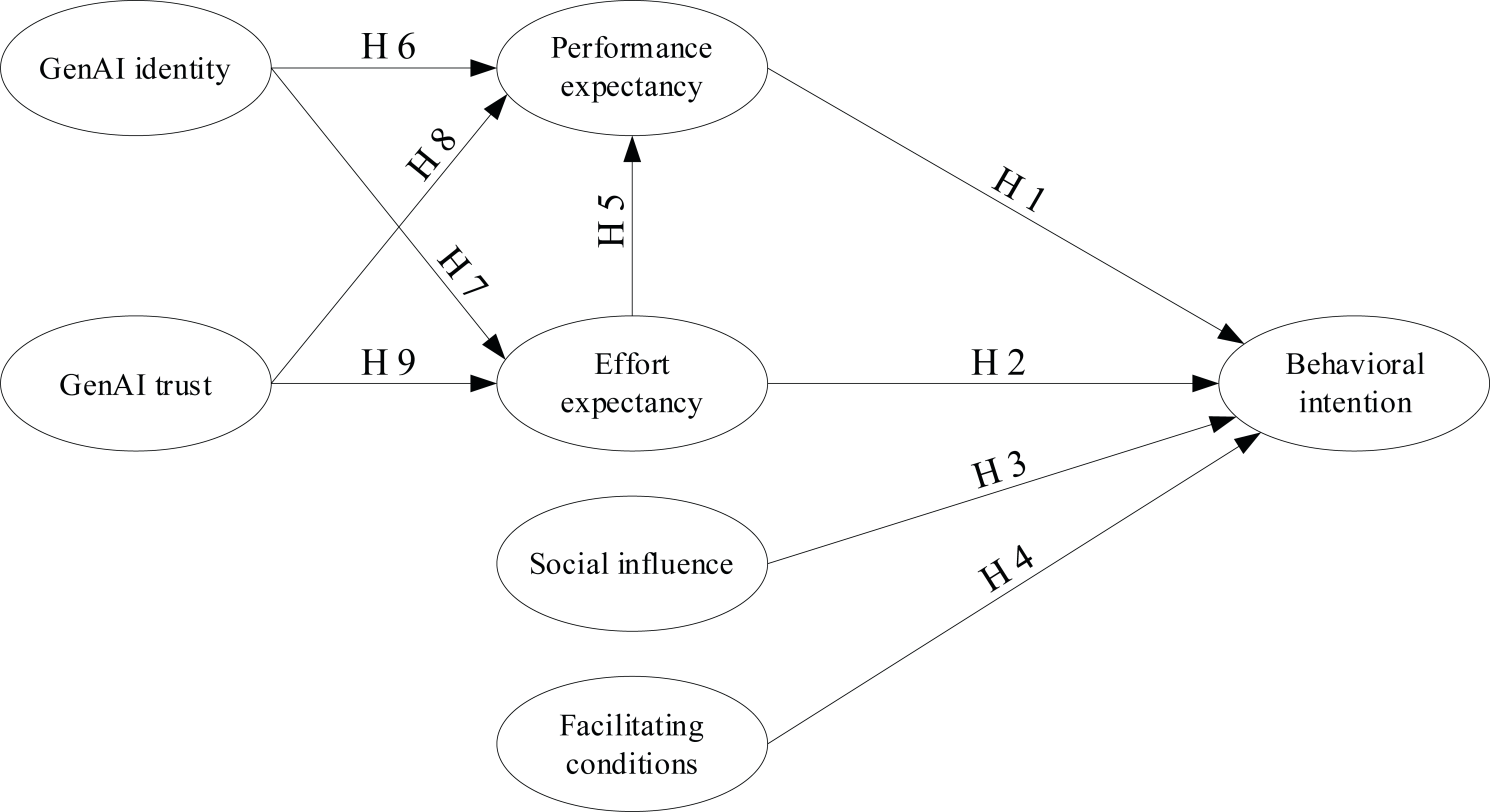
The conceptual model.

According to the UTAUT model, behavioral intention is determined by the direct effects of four core constructs (i.e., performance expectancy, effort expectancy, social influence, and facilitating conditions). This study simultaneously incorporated GenAI identity and trust into the UTAUT model to discuss adult learners’ behavioral intention toward GenAI usage. As presented by Figure 1, the extended UTAUT model consists of seven constructs and nine causal relationships. The relationships between the constructs (indicated by arrows) represent the hypotheses proposed in this study.

## Research methods

### Data collection

The survey data for this study were collected from adults aged 50 and above from different types of groups in central Taiwan, focusing on those who use GenAI tools for learning in their workplaces and daily lives. A questionnaire survey was employed to collect data within the defined research scope, and the instruments were administered through an online questionnaire and a survey distribution platform. The questionnaire was accompanied by a statement of the research purpose and an information sheet, informing all participants that their data would be kept strictly confidential and that anonymity and privacy would be fully ensured. Participants completed the questionnaire after fully understanding the research purpose, the voluntary nature of their participation, and the manner in which their data would be used. A total of 832 responses were initially collected in this survey. After incomplete responses and responses with patterned answering were excluded, 718 valid responses remained for analysis. This sample size meets the minimum requirement recommended by Hair et al. (2021) for PLS-SEM analysis, which suggests at least 10 observations per variable (Hair et al., 2021). Accordingly, the study was able to proceed to the subsequent stages of analysis.

### Measure development

This study’s survey instruments were carefully designed to ensure content validity, construct consistency, and measurement reliability. All measurement items were adapted from previously validated scales reported in the literature on GenAI, information systems, and AI. To ensure content validity, an expert panel of five scholars specializing in GenAI, information and technology management, and questionnaire design was convened to review the initially developed measurement items. The expert panel revised item wording, removed redundant content, and confirmed the operational definitions of each construct and their corresponding measurement items

The final questionnaire consisted of three parts. The first part provided an overview of the study, including its objectives and the implementation process. The second part primarily collected participants’ demographic statistical information, including gender, age, education level, average monthly income, and current occupational industry, which were rated using a nominal and ordinal scale. The third part comprised 5-point Likert-styled items aimed at collecting the adult participants’ responses to individual constructs of the research model, including those from the UTAUT model (i.e., performance expectancy, effort expectancy, social influence, facilitating conditions, behavioral intention), GenAI identity, and GenAI trust. Except for GenAI identity, which was measured using nine items, all other constructs were evaluated using four items. All items were rated using a 5-point Likert scale ranging from 1 (strongly disagree) to 5 (strongly agree). The questionnaire was written in Chinese to allow participants to answer in the language with which they were most familiar. By integrating multiple validated scales, this study effectively measured the specific performance of individual constructs, thereby ensuring the scientific rigidness and reliability of the findings.

Items for the UTAUT model (i.e., performance expectancy, effort expectancy, social influence, facilitating conditions, and behavioral intention) were developed based on Venkatesh et al. (2003). Previous studies (Chao, 2019; Fareed and Kirkil, 2025; Jin and Rui, 2026; Su and Chao, 2022; Wang et al., 2025; Xiaomin et al., 2025; Yakubu et al., 2025) were referred to for revision purposes. Items for GenAI identity were developed with reference to the research design of previous studies (Alahmad and Robert, 2020; Cao et al., 2023; Nash, 2024). Items for GenAI trust were developed according to the constructs in recent studies related to perceived AI trust (Tanantong and Wongras, 2024; Zhang and Yu, 2025; Zhang et al., 2025). Subsequently, a pilot study was conducted among 77 individuals from Taichung, Taiwan, to test the internal consistency of the measurement items. The results indicated that the Cronbach’s alpha values of all constructs met the recommended threshold of 0.70 suggested by Hair et al. (2010) confirming acceptable reliability. Based on the results of the pilot study, a final formal questionnaire was confirmed with 33 measurement items.

## Empirical analysis

This study employed Structural Equation Modeling (SEM) to examine the causal pathways among variables. Data analysis was conducted using SPSS 24.0 and Smart PLS 4.0. SPSS 24.0 was employed to perform descriptive statistical analysis of participants’ demographic information, levels of individual constructs, and correlations between the constructs. Smart PLS 4.0 was used to construct and evaluate the structural equation model, as well as to perform model fit assessment and path analysis.

### Participants’ sociodemographic characteristics

Questionnaire data from 718 adult learners aged 50 and above from central Taiwan were collected in this study. The participants’ demographic statistics were presented in Table 1. Among the 718 participants, 389 were female (54.2%), and 329 were male (45.8%). The participants were diversely distributed in terms of age, with those aged 50–59 being the predominant group (58.6%, *n* = 421), followed by those aged 60–69 (36.9%, *n* = 265) and those aged 70 and above (4.5%, *n* = 32). In terms of education level, 60.4% of the participants (*n* = 433) held a college or university degree, followed by those with a senior/vocational high school diploma (21.3%, *n* = 153). Regarding monthly income, 51.1% of the participants (*n* = 367) had a monthly income of NT$40,001–60,000, followed by a monthly income ranging from NT$20,001 to 40,000 (32.9%, *n* = 236). In terms of the current occupational industry, nearly half of the participants were employed in the healthcare and social work sector (49.8%, *n* = 358).

**TABLE 1:** Demographic details of respondents (*N* = 718).

Factor/Level	N	%	Factor/Level	N	%
*Gender*	329	45.8	*Age*	421	58.6
Male			50–59		
Female	389	54.2	60–69	265	36.9
Monthly income	67	9.3	≦ 70	32	4.5
< 20,000			Formal education		
20,001–40,000	236	32.9	Elementary school and below	42	5.8
40,001–60,000	367	51.1	Junior high school	57	7.9
60,001–80,000	34	4.7	Senior/vocational high school	153	21.3
80,001–100,000	10	1.4	College and university	433	60.4
> 100,001	4	0.6	Graduate institute and above	33	4.6
Current occupational industry					
Military personnel, civil servants, and public school teachers	54	7.5			
Manufacturing, information, and technology	36	5.0			
Finance and insurance	25	3.5			
Service sector	78	10.9			
Healthcare and social work	358	49.8			
Homemaker	38	5.3			
Retiree	51	7.1			
Others	78	10.9			

### Common method bias

As with all self-reported research, common method bias (CMB) may arise from multiple sources. To mitigate CMB, the questionnaire in this study first employed procedural remedies, including grouping unrelated constructs and randomly ordering items to create psychological separation. Second, Harman’s single-factor test was conducted on the seven latent variables in the research model to assess the severity of CMB (Podsakoff et al., 2003). The results showed that the seven latent variables explained 44.34% of the total variance, which is below the 50% threshold (Podsakoff et al., 2003; Harman, 1976) indicating that CMB is not a serious concern in this study. About the variance inflation factor (VIF) values, all constructs VIF values ranged from 1.189 to 2.553, which are below the threshold value of 3.3 (Kock, 2015; Podsakoff et al., 2003; Wang and Zhang, 2026). In addition, the f^2^ effect sizes indicated that the effects of EE on BI and PE on BI were negligible, with f^2^ values of 0.010 and 0.000, respectively. By contrast, the f^2^ values for the remaining relationships were all above 0.02, suggesting that these effects met at least the criterion for a small effect size (Podsakoff et al., 2003; Wang and Zhang, 2026).

### Measurement model analysis

First, both the reliability and validity of the measurement model were examined. Table 2 presents the analytical results, including the number of items, factor loadings, Cronbach’s a coefficients, composite reliability (CR), and average variance extracted (AVE). The results indicated that all items have factor loadings above the 0.7 level and are statistically significant (*p* < 0.05) (Hair et al., 2010). All constructs exhibit Cronbach’s α and CR values exceeding the recommended benchmark of 0.7, demonstrating strong internal consistency and satisfactory reliability. Furthermore, the AVE values for all constructs are greater than 0.5, meeting the criteria suggested by Hair et al. (2010) and confirming that each construct explains more than half of the variance in its indicators.

**TABLE 2:** Summary table of the reliability of different variables.

Construct	Number of items	Item loading	Cronbach’s α	rho_α	CR	AVE
GenAIID	9	0.711–0.928	0.953	0.983	0.957	0.715
GenAITR	4	0.744–0.933	0.909	0.987	0.935	0.783
PE	4	0.791–0.906	0.883	0.942	0.917	0.734
EE	4	0.890–0.957	0.941	0.950	0.958	0.850
SI	4	0.789–0.858	0.865	0.869	0.908	0.712
FC	4	0.799–0.927	0.880	0.907	0.916	0.732
BI	4	0.760–0.938	0.902	0.928	0.932	0.774

GenAIID, Generative AI identity; GenAITR, Generative AI trust; PE, Performance Expectancy; EE, Effort Expectancy; SI, Social Influence; FC, Facilitating Conditions; BI, Behavioral Intention; AVE, Average Variance Extracted; CR, Composite Reliability.

Discriminant validity analysis was performed using the criteria proposed by Fornell and Larcker (1981) which require that the square root of the AVE for each construct exceed its correlation coefficients with other constructs. In addition, all heterotrait–monotrait (HTMT) ratios were below the recommended threshold of 0.9, indicating adequate discriminant validity among the constructs (Fornell and Larcker, 1981; Henseler et al., 2015). As shown in Table 3, the square roots of AVE (reported on the diagonal) are greater than the inter-construct correlations (off-diagonal elements). Moreover, the highest HTMT value is 0.867, which is below the critical value of 0.9 (Henseler et al., 2015). Thus, these results demonstrate that the measurement model exhibits satisfactory reliability, convergent validity, and discriminant validity, providing a solid foundation for subsequent structural model analysis.

**TABLE 3:** The HTMT and correlations of the variables.

Construct	1.	2.	3.	4.	5.	6.	7.
1. GenAIID	**0.846**	0.348	0.533	0.585	0.411	0.461	0.270
2. GenAITR	0.331	**0.885**	0.431	0.408	0.605	0.448	0.496
3. PE	0.511	0.397	**0.857**	0.564	0.723	0.690	0.544
4. EE	0.569	0.391	0.531	**0.922**	0.692	0.799	0.592
5. SI	0.379	0.532	0.635	0.629	**0.844**	0.745	0.867
6. FC	0.434	0.414	0.617	0.737	0.650	**0.856**	0.708
7. BI	0.256	0.443	0.501	0.560	0.771	0.624	**0.880**

The bold diagonal value represents the square root of AVE; above the diagonal is the HTMT value, and below the diagonal line is the correlation coefficient between constructs.

### Structural model analysis

**FIGURE 2:**
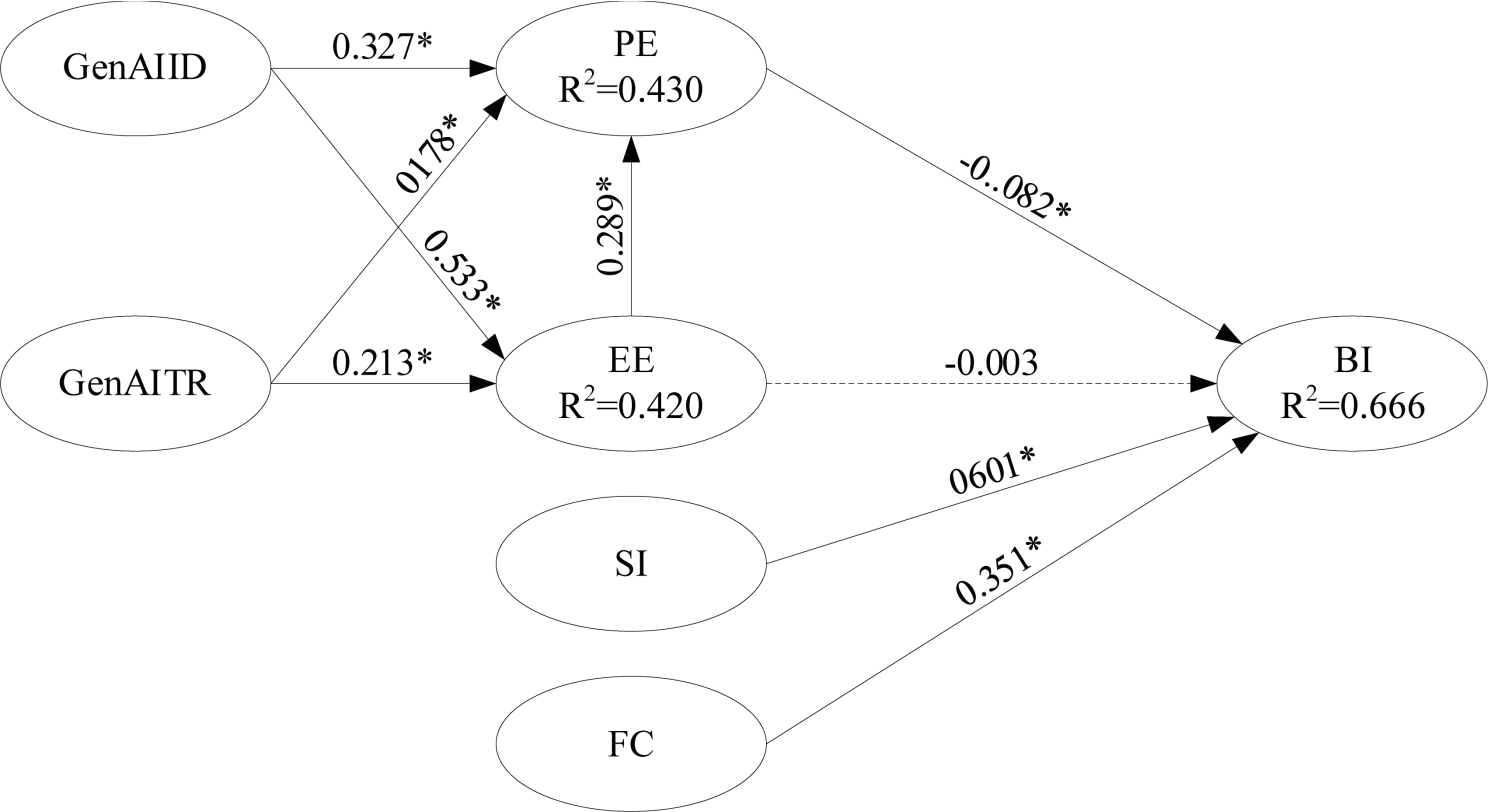
Research model diagram. *p < 0.05.

After the reliability and validity of the measurement model (external model) was validated, PLS-SEM was employed to evaluate the relationships between the latent constructs of the structural model (internal model). Bootstrapping with 5,000 resamples was conducted on the data from 718 participants to test the hypotheses. The results are presented in Figure 2 and Table 4.

**TABLE 4:** Verification results of hypotheses.

Hypothesis	Relationships between variables	Standardized coefficient	t-statistic	Test results
H 1	PE → BI	-0.082*	2.095	Rejected
H 2	EE → BI	-0.003	0.093	Rejected
H 3	SI → BI	0.601*	14.820	Supported
H 4	FC → BI	0.351*	12.173	Supported
H 5	EE → PE	0.289*	7.556	Supported
H 6	GenAIID → PE	0.327*	9.824	Supported
H 7	GenAIID → EE	0.533*	20.044	Supported
H 8	GenAITR → PE	0.178*	4.123	Supported
H 9	GenAITR → EE	0.213*	7.509	Supported

*p < 0.05.

The structural model was evaluated by path coefficients (β), *t*-values, and *p*-values. The model explains 66.6% of the variance in BI (*R*^2^ = 0.666), 43.0% of the variance in PE (*R*^2^ = 0.430), and 42.0% of the variance in EE (*R*^2^ = 0.420), which are all higher than the 0.26 threshold recommended by Hair et al. (2010), indicating that the model has a substantially high explanatory power (see Figure 2). The Q^2^ values of all constructs are above zero (from 0.375 to 0.663). This result suggests that the proposed model demonstrates adequate predictive relevance and is good fitted for this study (Hair et al., 2019; Wang and Zhang, 2026).

The results of hypothesis testing are presented in Figure 2 and Table 4. These results indicate that SI (H3, β = 0.601, *t* = 14.820) and FC (H4, β = 0.351, *t* = 12.173) exhibit significantly positive effects on the BI of GenAI usage, and SI is a significant influencing factor. Thus, Hypotheses 3 and 4 are supported. By contrast, PE (H1, β = –0.082, *t* = 2.095) shows significant negative effects on the BI of GenAI usage, and EE (H2, β = –0.003, *t* = 0.093) exhibits no influence on such BI. Thus, Hypotheses 1 and 2 are rejected. EE (H5, β = 0.289, *t* = 7.556) exhibits significantly positive effects on PE. Therefore, Hypothesis 5 is supported. In addition, this study found that GenAIID and GenAITR are significant antecedents of PE and EE. GenAIID exhibits significantly positive effects on PE (H6, β = 0.327, *t* = 9.824) and EE (H7, β = 0.533, *t* = 20.044). GenAITR shows significantly positive effects on PE (H8, β = 0.178, *t* = 4.123) and EE (H9, β = 0.213, *t* = 7.509). Therefore, Hypotheses 6, 7, 8 and 9 are supported. Figure 2 and Table 4 demonstrate the results of hypothesis testing for the research model. In addition, regarding the f^2^ effect sizes, the effects of EE on BI and PE on BI were negligible, with f^2^ values of 0.010 and 0.000, respectively. In contrast, the f^2^ values for the relationships among the other variables were all greater than 0.02, indicating that their effect sizes reached at least the threshold for a small effect (Hair et al., 2019; Wang and Zhang, 2026).

### Multi-group analysis

This study adopted PLS-multi-group analysis (PLS-MGA) to examine the structural differences across participant groups under an identical model estimation framework (Hair et al., 2021, Henseler et al., 2016). Specifically, the analysis assesses whether statistically significant differences exist in the structural path coefficients between group-specific models (e.g., male versus female participants) (Hair et al., 2021). The relevant statistical results and path comparisons are presented in Table 5.

**TABLE 5:** MGA-parametric test of gender differences in the research model.

	Gender				
Path relationship	Male *N* = 329	Female *n* = 389	Path coefficients (male vs. female)	*t*-value (male vs. female)	*p*-value (male vs. female)
PE → BI	-0.170	0.106*	-0.276*	3.015	0.003
EE → BI	0.591*	-0.041	0.632*	6.399	0.000
SI → BI	0.707*	0.425*	0.283*	3.239	0.001
FC → BI	-0.274*	0.467*	-0.741*	7.276	0.000
EE → PE	0.415*	0.143*	0.273*	3.376	0.001
GenAIID → PE	0.286*	0.419*	-0.133	1.928	0.054
GenAIID → EE	0.437*	0.583*	-0.146*	2.425	0.016
GenAITR → PE	0.292*	-0.011	0.303*	2.711	0.007
GenAITR → EE	0.311*	0.216*	0.095	1.375	0.169

*p < 0.05.

This study used the bootstrapping procedure of multi-group analysis (MGA) to compare differences in the estimated structural path coefficients between male and female participants, and further evaluated whether these differences reached statistical significance (*p* < 0.05). Table 5 presents the results of the MGA, indicating significant differences between male and female participants in diverse structural paths. This test is of critical importance, as it allows us to determine whether the structural model operates consistently and exhibits homogeneity across different groups.

The analytical results demonstrated that male and female participants differed in the influence of each construct on BI. In the path from PE to BI, the effect was significant only in the female participant sample (β = 0.106, *p* < 0.05), whereas it was not significant in the male participant sample (β = –0.170, *p* > 0.05), and the difference in path coefficients between the two groups reached statistical significance. In the path from FC to BI, the effect was significant in both groups (male: β = –0.274, *p* < 0.05; female: β = 0.467, *p* < 0.05), and the difference in path coefficients between the two groups also reached statistical significance (*p* < 0.05). In the path from GenAIID to EE, the effect was significant in both groups (male: β = 0.437, *p* < 0.05; female: β = 0.583, *p* < 0.05), and the difference in path coefficients between the two groups exhibited statistical significance (*p* < 0.05). These results suggested the effects along the relevant paths were stronger among female participants.

Conversely, in the path from EE to BI, the effect was significant only in the male participant sample (β = 0.591, *p* < 0.05), whereas it was not significant in the female participant sample (β = –0.041, p > 0.05), and the difference in path coefficients between the two groups reached statistical significance. In the path from SI to BI, the effect was significant in both groups (male: β = 0.707, *p* < 0.05; female: β = 0.425, *p* < 0.05), and the difference in path coefficients between the two groups also reached statistical significance (*p* < 0.05). In the path from EE to PE, the effect was significant in both groups (male: β = 0.415, *p* < 0.05; female: β = 0.143, *p* < 0.05), and the difference in path coefficients between the two groups reached statistical significance (*p* < 0.05). In the path from GenAITR to PE, the effect was significant only in the male participant sample (β = 0.292, *p* < 0.05), whereas it was not significant in the female participant sample (β = -0.011, *p* > 0.05), and the difference in path coefficients between the two groups reached statistical significance (*p* < 0.05). Overall, the effects along these structural paths were stronger among male participants.

In addition, while significant effects were identified in both groups in the paths from GenAIID to PE (male: β = 0.286, *p* < 0.05; male: β = 0.419, *p* < 0.05) and from GenAITR to EE (male: β = 0.311, *p* < 0.05; female: β = 0.216, *p* < 0.05), no significant differences were observed in the strength of effects between the two groups. Overall, the findings indicated that the structural model in this study demonstrated partial invariance across gender groups, suggesting that certain structural relationships differed by gender.

## Discussion

This study aimed to explore key determinants of adult learners’ BI s of adopting GenAI in education and learning activities through an extended UTAUT model, with a research sample comprising adults aged 50 and above in central Taiwan. It examined the role of GenAIID, GenAITR, PE, EE, SI, and FC in shaping adult learners’ BI of using GenAI.

Overall, SI and FC are crucial determinants of adult learners’ BI of using GenAI. By contrast, PE exhibits a negative influence on BI, and EE shows no significant influence. Specifically, SI is the most important influencing factor of adult learners’ BI to use GenAI, which suggests that adult learners believe that influence from others (e.g., colleagues, supervisors, friends, or families) plays a key role in enhancing the BI of GenAI adoption. This finding is consistent with previous studies and further validates the important role of SI in technology acceptance (Li and Wei, 2025; Wang et al., 2025; Xiaomin et al., 2025; Yakubu et al., 2025; Kim et al., 2024). This finding also indicates that positive opinions and support from important others play a crucial role in the successful adoption of new technology for adult learners.

The second most significant predictive variable of BI is FC. This finding aligns with previous studies (Kim et al., 2024; Li and Wei, 2025; Tanantong and Wongras, 2024; Xiaomin et al., 2025). GenAI is an AI system that primarily adopts the form of conversational human-machine interaction, which transforms traditional information search and knowledge production patterns (Zhang and Yu, 2025). When adult learners feel familiar with GenAI and have adequate resources, supportive mechanisms, and relevant knowledge to use the technology, their willingness to use GenAI will increase, which plays a significant role in facilitating usage intention. In addition, we found that PE exhibits negative effects on BI toward GenAI. This is inconsistent with previous studies (Gazit et al., 2026; Yakubu et al., 2025; Xiaomin et al., 2025). This finding suggests that, as adult learners often have stable and mature learning strategies, when GenAI is expected to “significantly enhance” learning performance, exceedingly high PE may cause doubts about its reliability, controllability, and educational appropriateness, thereby reducing the usage intention.

It is noteworthy that EE does not exhibit statistically significant effects on the BI of GenAI adoption. This finding diverges from the classical hypothesis of the UTAUT model, which posits that EE positively influences users’ BI. The present study’s finding indicates that EE concerning generative AI does not determine BI. This is likely due to the rapid and widespread application of GenAI in recent years. Since the launch of ChatGPT by OpenAI in 2022, various GenAI tools have been gradually introduced, contributing to significant worldwide growth in the application and development of GenAI. The growth of GenAI has also consistently driven innovation in various industries, with its scope of application and potential value steadily expanding across diverse domains including education. In this context, adult learners are now broadly exposed to and familiar with multiple GenAI tools. Moreover, the proliferation of instructional videos and usage guides on online platforms has lowered users’ perceived operational complexity at the psychological level. Consequently, the participants may generally perceive the use of GenAI as requiring little effort. Similar findings are also reported in a study on older adults’ use of AI voice assistants (Li and Wei, 2025).

Regarding the constructs incorporated into the UTAUT model, this study found that GenAIID and GenAITR are significant antecedent factors of PE and EE. As far as we know, previous research has not yet systematically examined the influence of GenAIID and trust on PE and EE. This research gap is addressed by the present study, which constitutes one of this study’s primary theoretical contributions. First, this study enriches the literature on AI identity and the application of AI in educational scenarios by proposing a new perspective on the consequence of GenAIID. This study’s findings also suggest that when adult learners have relatively high levels of identity with GenAI and view GenAI as part of their identity as learners, they are more likely to perceive the technology as effectively enhancing teaching quality, learning effectiveness, and work efficiency, thereby improving their PE. In addition, such identity helps to alleviate psychological resistance against the complexity and uncertainties of technology, and leads adult learners to subjectively perceive the effort required to operate and learn generative AI as relatively low, thereby enhancing their EE. Furthermore, this study found that GenAITR is a key external factor that influences PE and EE in the UTAUT model. This finding indicates that higher levels of trust in GenAI directly enhance adult learners’ performance in learning activities and improve their perceived ease of use concerning GenAI. This finding is also consistent with previous studies (Khalilzadeh et al., 2017; Tanantong and Wongras, 2024) which demonstrates that trust is an important antecedent factor of PE and EE.

Previous studies have indicated that gender differences may influence individuals’ use and acceptance of information and communication technology (ICT) (Møgelvang et al., 2024; Qazi et al., 2022). In the context of GenAI applications, Møgelvang et al. (2024) found that, compared with women, men are more likely to perceive GenAI chatbots as having instrumental value and to show greater interest in the application of GenAI chatbots. This finding suggests that if gender differences exist in adult learners’ perceptions and use of GenAI, such differences may further influence their behavioral intention to use GenAI. Therefore, educational institutions should pay attention to differences in GenAI use experiences among learners of different genders in order to reduce potential gender gaps that may arise in the process of technology application. Accordingly, this study treated gender as a moderating variable and conducted a group comparison between male and female learners using multi-group analysis (MGA) to examine the moderating effect of gender on the proposed research model. The results indicated that, compared to female adult learners, male adult learners exhibit differences in the coefficients of the paths from EE to BI, from SI to BI, from EE to PE, and from GenAITR to PE. These findings suggest that when male adult learners perceive GenAI as easy to use and helpful, and when their important others (e.g., families, friends, colleagues, or supervisors) support the adoption of GenAI, their BI to use GenAI will be enhanced accordingly. In addition, when they perceive GenAI as helpful and hold favorable attitudes toward and feel confident about GenAI, they are more convinced that the use of GenAI can enhance their learning performance. Moreover, compared to male learners, female learners exhibit difference in the coefficients of the paths from PE and FC to BI and from GenAITR to EE. This suggest that when female learners perceive GenAI as helpful to improve their learning performance, and when they can seek help from someone when encountering problems, their BI to use GenAI will be enhanced. In conclusion, gender has moderating effects on the research model. This finding extends the applicational scope of gender in the UTAUT model and the fields of adult learning and GenAI, contributing to the relevant literature.

## Implications

By incorporating GenAIID and trust into the UTAUT model, this study established a research model to explain adult learners’ BI of GenAI usage. It contributes to the current literature as follows: first, this study extended the UTAUT model to research on adult learning and the application of GenAI; second, it identified SI and facilitation conditions as important antecedent factors influencing adult learners’ BI of GenAI usage; third, it identified GenAIID and trust as significant predictive variables influencing PE and EE in the UTAUT model. Finally, multi-group analysis was employed to investigate the moderating effects of gender on the research model, thereby identifying differences in path coefficients across gender groups.

## Research limitations and recommendations for future research

This study introduced GenAIID and trust to the UTAUT model, and explored and validated key factors influencing adult learners’ BI of using GenAI. It enhanced the model’s explanatory power and offered significant practical implications. Nevertheless, this study still presents several limitations. First, this study employed a cross-sectional research design and was unable to examine the long-term causal relationships between constructs. Future research could use diverse research designs, such as longitudinal or experimental designs, to examine the dynamics of usage intention, thereby revealing the long-term effects of the influencing factors. Second, as the research data were mainly collected from central Taiwan, the results may reflect regional and cultural factors. Future research could expand the research area to other geographical regions and communities with diverse cultural backgrounds and age groups to replicate and test the model proposed by this study. These improvements will yield more comprehensive conclusions and enhance the universal applicability of the research results, providing valuable reference to the global promotion of GenAI application. Third, most participants in this study were recruited through online channels or questionnaire distribution platforms. The overall sample possessed a certain level of information competency, suggesting that it may be difficult to generalize the results to communities with lower levels of information competency. Future studies could employ diverse questionnaire distribution methods. Finally, this study’s data were mainly collected from a self-report questionnaire survey, which may have caused common method bias and overestimates of the relationships between constructs, although measures such as participant anonymity and use of neutral wording had been adopted to reduce bias.

## Conclusion

By integrating GenAIID and trust into the UTAUT model, this study comprehensively examined determinants of adult learners’ BI to use GenAI. The findings suggest that SI is the most significant influencing factor of such intention, and FC are the second most significant influencing factor. However, PE exhibits negative effects and EE shows no statistically significant effects on BI. These results differ from the central hypothesis of the UTAUT that both PE and EE have positive effects on BI. Overall, this study demonstrates that most constructs of the UTAUT model still possess theoretical explanatory power in the contexts of GenAI usage. It provides a significant contribution to both the theoretical advancement and empirical examination of the UTAUT model within emerging technology contexts.

In addition, this study introduced GenAIID and trust into the UTAUT model to gain a deeper understanding of adult learners’ BI of GenAI usage. The results demonstrated that GenAIID and trust are significant external variables influencing PE and EE. Finally, a multi-group analysis of gender differences identified significant differences across genders in most structural paths of the model, except for the paths from GenAIID to PE and from GenAITR to EE. In conclusion, although few studies have incorporated GenAIID and trust into the UTAUT model, this study addresses this research gap and advances theoretical understanding of adult learners’ technology adoption in generative AI settings, while offering valuable and original empirical contributions to this emerging field.

## Data Availability

The raw data supporting the conclusions of this article will be made available by the authors, without undue reservation.

## References

[B1] Agarwal R. Karahanna E. (2000). Time flies when you’re having fun: cognitive absorption and beliefs about information technology usage1. *MIS Q.* 24 665–694. 10.2307/3250951

[B2] Alahmad R. Robert L. (2020). Artificial intelligence (AI) and IT identity: antecedents identifying with AI applications. *arXiv [Preprint].* 10.48550/arXiv.2005.12196

[B3] Amoozegar A. Abdelmagid M. Anjum T. (2024). Course satisfaction and perceived learning among distance learners in Malaysian research universities: the impact of motivation, self-efficacy, self-regulated learning, and instructor immediacy behaviour. *Open Lean.* 39 387–413. 10.1080/02680513.2022.2102417

[B4] Aron A. Aron E. N. Norman C. (2003). “Self-expansion model of motivation and cognition in close relationships and beyond,” in *Blackwell Handbook of Social Psychology: Interpersonal Processes* (Los Angeles, CA: University of California), 478–501.

[B5] Burke P. J. Stets J. E. (2009). *Identity Theory.* New York, NY: Oxford University Press.

[B6] Cao L. Chen C. Dong X. Wang M. Qin X. (2023). The dark side of AI identity: investigating when and why AI identity entitles unethical behavior. *Comput. Hum. Behav.* 143:107669. 10.1016/j.chb.2023.107669

[B7] Carter M. Petter S. Grover V. Thatcher J. B. (2020). Information technology identity: a key determinant of IT feature and exploratory usage. *MIS Q.* 44 983–1022. 10.25300/MISQ/2020/14607

[B8] Chao C. M. (2019). Factors determining the behavioral intention to use mobile learning: an application and extension of the UTAUT model. *Front. Psychol.* 10:1652. 10.3389/fpsyg.2019.01652 31379679 PMC6646805

[B9] Chi O. H. Chi C. G. Gursoy D. Nunkoo R. (2023). Customers’ acceptance of artificially intelligent service robots: the influence of trust and culture. *Int. J. Inf. Manage.* 70:102623. 10.1016/j.ijinfomgt.2023.102623

[B10] Chonsalasin D. Champahom T. Limpasirisuwan N. Jomnonkwao S. Ratanavaraha V. (2025). Urban-rural differences in electric vehicle adoption intentions: integrated TAM, TPB, UTAUT with environmental identity. *Civ. Eng. J.* 11 1891–1923. 10.28991/CEJ-2025-011-05-010

[B11] Choung H. David P. Ross A. (2023). Trust in AI and its role in the acceptance of AI technologies. *Int. J. Hum. Comput. Interact.* 39 1727–1739. 10.1080/10447318.2022.2050543

[B12] Fareed A. S. Kirkil G. (2025). Integrating technology acceptance model with UTAUT to increase the explanatory power of the effect of HCI on Students’ intention to use E-Learning system and perceive success. *IEEE Access* 13 20720–207390. 10.1109/ACCESS.2025.3534740

[B13] Fornell C. Larcker D. F. (1981). Evaluating structural equation models with unobservable variables and measurement error. *J. Mark. Res.* 18 39–50. 10.1177/002224378101800104

[B14] Fu M. Han Y. Chen Y. Zhang J. (2025). Exploring user intentions for Virtual Memorialization: an integration of TAM and social identity in immersive environments. *Appl. Sci.* 15:11240. 10.3390/app152011240

[B15] Gazit T. Eitan T. Gal L. Gradovitch N. (2026). Adoption of generative AI technologies: insights from the UTAUT2 model, personality characteristics, and behavioural factors. *J. Comput. Assist. Learn.* 42:e70162. 10.1002/jcal.70162

[B16] Gorges J. (2025). Global motivation to learn and adult learning: a nomological network analysis and a four-year longitudinal study. *Learn. Individ. Differ.* 123:102763. 10.1016/j.lindif.2025.102763

[B17] Green D. N. (2024). Adult learners: connecting andragogy and transformational leadership in the classroom. *Bus. Ethics Leadersh.* 8 137–147. 10.61093/bel.8(4).137-147.2024

[B18] Hair J. F. Jr. Hult G. T. M. Ringle C. M. Sarstedt M. Danks N. P. Ray S. (2021). *Partial Least Squares Structural Equation Modeling (PLS-SEM) using R: A Workbook.* Cham: Springer Nature, 197.

[B19] Hair J. F. Black W. C. Babin B. J. Anderson R. E. (2010). *Multivariate Data Analysis: A Global Perspective*, 7th (Ed.) Edn. New York, NY: MacMillan.

[B20] Hair J. F. Risher J. J. Sarstedt M. Ringle C. M. (2019). When to use and how to report the results of PLS-SEM. *Eur. Bus. Rev.* 31 2–24. 10.1108/EBR-11-2018-0203

[B21] Harman H. H. (1976). *Modern Factor Analysis.* Chicago, IL: University of Chicago press.

[B22] Henseler J. Hubona G. Ray P. A. (2016). Using PLS path modeling in new technology research: updated guidelines. *Ind. Manage. Data Syst.* 116 2–20. 10.1108/IMDS-09-2015-0382

[B23] Henseler J. Ringle C. M. Sarstedt M. (2015). A new criterion for assessing discriminant validity in variance-based structural equation modeling. *J. Acad. Mark. Sci.* 43 115–135. 10.1007/s11747-014-0403-8

[B24] Jin H. Rui Y. (2026). Gen AI literacy and acceptance among EFL teachers: an exploration through the UTAUT model and self-determination theory. *Eur. Eur. J. Educ.* 61:e70364. 10.1016/j.ssaho.2026.102457

[B25] Khalilzadeh J. Ozturk A. B. Bilgihan A. (2017). Security-related factors in extended UTAUT model for NFC based mobile payment in the restaurant industry. *Comput. Hum. Behav.* 70 460–474. 10.1016/j.chb.2017.01.001

[B26] Kim J. Yu S. Detrick R. Lin X. Li N. (2025). Designing AI-powered learning: Adult learners’ expectations for curriculum and human-AI interaction. *Educ. Tech. Res. Dev.* 73 3397–3421. 10.1007/s11423-025-10549-z

[B27] Kim Y. Blazquez V. Oh T. (2024). Determinants of generative AI system adoption and usage behavior in Korean companies: applying the UTAUT model. *Behav. Sci.* 14:1035. 10.3390/bs14111035 39594336 PMC11591487

[B28] Kock N. (2015). Common method bias in PLS-SEM: a full collinearity assessment approach. *Int. J. e-Collab.* 11 1–10. 10.4018/ijec.2015100101

[B29] Kolesar M. B. Galbraith R. W. (2000). A services-marketing perspective on e-retailing: implications for e-retailers and directions for further research. *Internet Res.* 10 424–438.

[B30] Li H. Wei X. (2025). Factors influencing older adults’ adoption of AI voice assistants: extending the UTAUT model. *Front. Psychol.* 16:161868. 10.3389/fpsyg.2025.1618689 41293105 PMC12641254

[B31] Liu X. He X. Wang M. Shen H. (2022). What influences patients’ continuance intention to use AI-powered service robots at hospitals? The role of individual characteristics. *Technol. Soc.* 70:101996. 10.1016/j.techsoc.2022.101996

[B32] Liu X. S. Yi X. S. Wan L. C. (2022). Friendly or competent? The effects of perception of robot appearance and service context on usage intention. *Ann. Touris. Res.* 92:103324.

[B33] Loeng S. (2018). Various ways of understanding the concept of andragogy. *Cogent Educ.* 5:1496643.

[B34] Lv X. Yang Y. Qin D. Cao X. Xu H. (2022). Artificial intelligence service recovery: the role of empathic response in hospitality customers’ continuous usage intention. *Comput. Hum. Behav.* 126:106993.

[B35] McKenna K. Gupta K. Kaiser L. Lopes T. Zarestky J. (2020). Blended learning: balancing the best of both worlds for adult learners. *Adult Learn.* 31 139–149. 10.1177/1045159519891997

[B36] Møgelvang A. Bjelland C. Grassini S. Ludvigsen K. (2024). Gender differences in the use of generative artificial intelligence chatbots in higher education: characteristics and consequences. *Educ. Sci.* 14:1363. 10.3390/educsci14121363

[B37] Nash K. (2024). Exploring the impact of self-concept and IT identity on social media influencers’ behavior: a focus on young adult technology features utilization. *Int. J. Hum. Comput. Interact.* 40 6941–6952. 10.1080/10447318.2023.2271235

[B38] Podsakoff P. M. MacKenzie S. B. Lee J. Y. Podsakoff N. P. (2003). Common method biases in behavioral research: a critical review of the literature and recommended remedies. *J. Appl. Psychol.* 88:879. 10.1037/0021-9010.88.5.879 14516251

[B39] Qazi A. Hasan N. Abayomi-Alli O. Hardaker G. Scherer R. Sarker Y. et al. (2022). Gender differences in information and communication technology use & skills: a systematic review and meta-analysis. *Educ. Inf. Technol* 27 4225–4258. 10.1007/s10639-021-10775-x 34697533 PMC8528947

[B40] Reychav I. Beeri R. Balapour A. Raban D. R. Sabherwal R. Azuri J. (2019). How reliable are self-assessments using mobile technology in healthcare? The effects of technology identity and self-efficacy. *Comput. Hum. Behav.* 91 52–61. 10.1016/j.chb.2018.09.024

[B41] Rodrigo C. Iniesto F. Garcia-Serrano A. (2024). Applying andragogy for integrating a MOOC into a formal online learning experience in computer engineering. *Heliyon* 10:e23493. 10.1016/j.heliyon.2023.e23493 38173478 PMC10761581

[B42] Song X. Zhang Y. Lu Z. Xu L. Shen H. (2026). Generative AI: a double-edged sword for creative thinking learning—evidence from facial expressions and fNIRS. *Comput. Educ.* 247:105578. 10.1016/j.compedu.2026.105578

[B43] Stets J. E. Biga C. F. (2003). Bringing identity theory into environmental sociology. *Sociol. Theor.* 21 398–423.

[B44] Su C. Y. Chao C. M. (2022). Investigating factors influencing nurses’ behavioral intention to use mobile learning: using a modified unified theory of acceptance and use of technology model. *Front. Psychol.* 13:673350. 10.3389/fpsyg.2022.673350 35651564 PMC9150498

[B45] Tanantong T. Wongras P. (2024). A UTAUT-based framework for analyzing users’ intention to adopt artificial intelligence in human resource recruitment: a case study of Thailand. *Systems* 12:28.

[B46] Thaiduong N. (2026). Public trust in AI: a dynamic social media view. *Soc. Sci. Humanit. Open* 13:102457. 10.1016/j.ssaho.2026.102457

[B47] Trang T. T. N. Thang P. C. Vo T. A. (2025). Moderating the AI revolution: perceived threat and generative AI in vietnamese hospitals. *Comput. Hum. Behav. Rep.* 19:100774. 10.1016/j.chbr.2025.100774

[B48] van der Stap N. van den Bogaart T. Rahimi E. van Ginkel S. Nelson T. Versendaal J. (2024). Supporting adult-learners’ online interaction in computer-mediated learning. *Comput. Hum. Behav. Rep.* 16:100551. 10.1016/j.chbr.2024.100551

[B49] Venkatesh V. Morris M. G. Davis G. B. Davis F. D. (2003). User acceptance of information technology: toward a unified view. *MIS Q.* 27 425–478. 10.2307/30036540

[B50] Wang X. Li M. Yao Y. Zhu X. (2025). A study of factors influencing Chinese design students’ adoption of AIGC tools. *IEEE Access* 13 117753–117770. 10.1109/ACCESS.2025.3582599

[B51] Wang X. Zhang L. J. (2026). What drives GenAI adoption in informal digital learning of English (IDLE)?: structural-configurational modelling of extended UTAUT2 with GenAI literacy. *J. Comput. Assist. Learn.* 42:e70217. 10.1002/jcal.70217

[B52] Xiaomin J. Kai C. Ping W. (2025). Adoption intention of generative artificial intelligence among Chinese college students: an extended TAM-UTAUT2 model from the four-helix perspective. *Front. Educ.* 10:1686408. 10.3389/feduc.2025.1686408

[B53] Yakubu M. N. David N. Abubakar N. H. (2025). Students’ behavioural intention to use content generative AI for learning and research: a UTAUT theoretical perspective. *Educ. Inf. Technol.* 30 17969–17994. 10.1007/s10639-025-13441-8

[B54] Zhang C. Hu M. Wu W. Kamran F. Wang X. (2025). Unpacking perceived risks and AI trust influences pre-service teachers’ AI acceptance: a structural equation modeling-based multi-group analysis. *Educ. Inf. Technol.* 30 2645–2672. 10.1007/s10639-024-12905-7

[B55] Zhang G. Yu T. (2025). Association between generative AI self-efficacy and generative AI acceptance: the mediating role of generative AI trust and the moderating role of generative AI risk perception. *Acta Psychol.* 261:105791. 10.1016/j.actpsy.2025.105791 41101128

